# High-Performance and Self-Powered Deep UV Photodetectors Based on High Quality 2D Boron Nitride Nanosheets

**DOI:** 10.3390/nano7120454

**Published:** 2017-12-19

**Authors:** Ali Aldalbahi, Manuel Rivera, Mostafizur Rahaman, Andrew F. Zhou, Waleed Mohammed Alzuraiqi, Peter Feng

**Affiliations:** 1 Department of Chemistry, College of Science, King Saud University, Riyadh 11451, Saudi Arabia; mrahaman@ksu.edu.sa (M.R.); walzurauqi@ksu.edu.sa (W.M.A.); 2 Department of Physics, University of Puerto Rico, San Juan, PR 00936-8377, USA; manuel.rivera3@upr.edu; 3 Department of Physics, Indiana University of Pennsylvania, Indiana, PA 15705, USA; fzhou@iup.edu

**Keywords:** deep ultraviolet, photodetector, boron nitride, nanosheets, self-powered, Schottky junction

## Abstract

High-quality two-dimensional (2D) crystalline boron nitride nanosheets (BNNSs) were grown on silicon wafers by using pulsed plasma beam deposition techniques. Self-powered deep ultraviolet (DUV) photodetectors (PDs) based on BNNSs with Schottky contact structures are designed and fabricated. By connecting the fabricated DUV photodetector to an ammeter, the response strength, response time and recovery time to different DUV wavelengths at different intensities have been characterized using the output short circuit photocurrent without a power supply. Furthermore, effects of temperature and plasma treatment on the induced photocurrent response of detectors have also been investigated. The experimental data clearly indicate that plasma treatment would significantly improve both induced photocurrent and response time. The BNNS-based DUV photodetector is demonstrated to possess excellent performance at a temperature up to 400 °C, including high sensitivity, high signal-to-noise ratio, high spectral selectivity, high speed, and high stability, which is better than almost all reported semiconducting nanomaterial-based self-powered photodetectors.

## 1. Introduction

The fabrication of novel deep ultraviolet (DUV) emitters and high-temperature deep-ultraviolet photodetectors have been major challenges in high-performance optoelectronic research. Recent developments in the exploitation of novel DUV emitter multilayer structures have been reported [1,2,3], based on hexagonal crystal boron nitride (hBN) and II–VI semiconductor heterostructures, tunnel injection of carriers, and polarization-induced p-type doping to potentially overcome the intrinsic problem of low free hole concentration in short-wavelength emitter systems. Similarly, multilayer structures are also widely employed for the development of different types of DUV photodetectors [4,5]. Several high-sensitivity, quick-response DUV photodetectors have been developed [6,7]. Comprehensive reviews of various types of wide band gap semiconductor (WBGS)-based DUV photodetectors have been presented by Sang [8], BenMoussa [9], and Goldsman [10], respectively. Generally, all of these WBGS-based ultra-violet (UV) photodetectors can be classified into the following four types: Schottky barrier, metal-semiconductor-metal diodes, photoconductors, and p-i-n photodiodes [11]. Most of the fabricated WBGS-based devices have been designed specifically for visible-blind UV light detection applications. The main focus of WBGS material research for these DUV photodetectors is on oxide (TiO_2_, ZnO) [12,13], nitride (AlN, GaN, BN) [14,15,16], SiC [17], and diamond [18,19] semiconducting materials, as well as various composites, such as AlGaN and InGaN, etc. [20,21] but most of them cannot be operated at extremely high temperatures up to 400 °C. Relatively, diamond has very high strength, excellent electrical properties, and high temperature stability, which makes it an attractive choice for high temperature. However, no ork related to self-powered diamond-based photodetectors (PDs) has been reported before. 

Boron nitride (BN) is one of the most important wide band gap materials for optoelectronic devices, which has received increasing attention due to its excellent chemical inertness, long-term stability when it is operated under high intensity UV light radiation at a high operating temperature [22,23]. It is known that the bulk BN is a direct band gap semiconductor with a band gap up to 5.97 eV. This makes it a good candidate for wide applications in detecting the DUV radiation without the need for solar rejection filters. In addition to solar-blind operation, BN-based photodetectors could achieve large gains and high signal-to-noise ratios.

Interestingly, the features of the very thin two-dimensional (2D) BN nanosheets (BNNSs) are slightly different to that of the BN films and bulk materials. Recent experimental research indicated that BNNSs have a variable band gap width that can be controlled with their sheet edge structures, sheet orientations, and sheet thickness (the numbers of atomic layers in a sheet). For example, an obvious red shift of DUV spectral lines was observed from experiments with a thin BNNS sample that showed a sharp cut-off wavelength down to 254 nm [24], shifting almost 8% from the cut-off wavelength of 230 nm of the bulk BN [25]. Experiments from photoluminescence spectral measurements indicated that energy band gaps of BNNSs can vary following their orientations in a wide range, even as low as 3 eV [26]. All these results suggested that 2D BN nanosheet materials could be more appropriate for applications of high-performance DUV photodetection [1,4,24], capable of having a good selectivity within the UV spectral range and sharp cut-off frequency, combined with the previously-mentioned benefits, such as endurance and chemical inertness. Our preliminary experimental data revealed that the thin BNNS-based photodetector has quicker/shorter response time than that of the thick BN-based detector, indicating its potential applications for high-speed electronic devices. However, the development of high-performance 2D BNNS UV photodetectors is challenging due to the 2D sheet material defects, and poorly-modeled device operation.

The present work extends the state-of-the-art in deep UV photodetectors by using the high-quality crystalline BNNSs to build a controllable Schottky barrier for self-powered high-performance (high-sensitivity, high-stability) DUV photodetectors operating at high temperature up to 400 °C. The experimental data clearly indicated that the obtained responsivity of the newly-designed BNNS-based PDs is much better than the traditional oxide semiconductor-based self-powered PDs. 

Furthermore, plasma treatment techniques are used in our work to further enhance the induced photocurrent response and performance of the prototypes. Since a Schottky barrier on very thin 2D BN nanosheets is built, fabrication of self-powered photodetector having a significantly low dark current becomes possible. Various new phenomena, including cut-off wavelength shift, thermal effect, and self-power capabilities, have been studied. The basic properties of the prototypes, such as response strength and sensitivity, response time and recovery time, repeatability, and stability before and after the plasma treatments, have been carefully studied. The newly-fabricated DUV photodetectors appear to have a very stable baseline and excellent repeatability. A responsivity up to 296 mA/W and a signal-to-noise ratio up to 350 have been achieved at zero bias. Even at a 400 °C operating temperature, the newly-fabricated prototypes still display good responsivity, high stability, and excellent repeatability.

## 2. Experimental Section

### 2.1. Synthesis and Basic Characterization of BNNSs

Several high-temperature DUV photodetectors based on wide band gap oxide semiconductors have been reported recently, but none of them can be operated at a temperature up to 200 °C [27,28]. A good performance of a DUV photodetector directly relies on its wide band gap width material quality, the device design, and fabrication. Our previous experiments have clearly indicated that high-quality BNNS is one of the ideal candidates for high-temperature DUV photodetectors. However, the serious diffusions in the poor quality of BNNSs caused at high operating temperatures would affect the properties of the electronic device. Consequently, the obtained electronics were not able to be operated at temperatures above 300 °C.

Successful syntheses of super-thin 2D boron nitride nanosheets have been carried out by many research groups based on various techniques, including chemical vapor deposition (CVD) [29,30], chemical blowing [31], chemical exfoliation [32,33], ball milling process [34], micromechanical cleavage [35], and liquid exfoliation [36], where the main method for achieving large BNNSs relies on CVD. However, the growth rate with CVD is relatively low [26,29,30], and it is also possible to have impurities because CVD precursors usually contain multiple component substances [37]. Furthermore, high temperatures up to 1000 °C in CVD process [26,30] would possibly vaporize the impurities inside the chamber, which would also result in internal stresses that might affect the crystalline BNNSs. 

In this present work, a short laser pulse produced plasma beam deposition technique is used, with which either contamination or internal stresses can be largely avoided during synthesis of crystalline BNNSs. A detailed description of pulsed laser-produced plasma beam deposition technique can be found in our previous paper [38]. Briefly, a high power CO_2_ laser beam (pulse energy: 5 J; pulse width: 2 µs; repetition frequency: 5 Hz) is focused onto a rotated hexagonal BN target with a laser power density around 2 × 10^8^ W/cm^2^ per pulse. Silicon wafers as substrates are used, and placed 4.5–5 cm away from the plasma source. The temperature of the substrate is 400 °C. The deposition durations are 5 and 50 min, respectively, for obtaining the desired thicknesses of the boron nitride samples. Normally, each sample consists of numerous BNNS clusters and each individual BNNS cluster is around 4–5 nm in thickness. The morphologies of the BNNSs are analyzed by using a scanning electron microscope (SEM) and a transmission electron microscopy (TEM). Physical properties are characterized by using the Raman spectrum and XRD, respectively. Finally, BNNS-based, self-powered DUV photodetectors were designed, fabricated, and tested.

### 2.2. SEM and TEM Analyses

**Figure 1:**
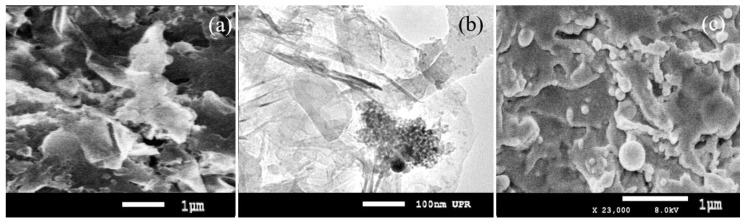
SEM images of BNNS samples prepared on Si substrates for different durations (**a**) 5, and (**c**) 50 min; and (**b**) TEM image of a selected surface area from the BNNS sample (Figure 1a).

Figure 1a,c show the typical SEM images of the BNNSs deposited on Si substrates with different deposition durations. The sheet structure in Figure 1a is clearly visible. Entire surfaces (1 × 1 cm) of the substrates are covered with BNNS clusters. The average size of a continuous BNNS is approximately 4–10 µm^2^. The surfaces of the samples appear quite rough, because each boron nitride nanosheet cluster in the sample has a random orientation. 

In order to understand the quality of the fabricated BNNSs, TEM is also employed for further structural characterizations, as shown in Figure 1b, where the ultra-thin BNNSs were collected by directly scratching of the BNNSs from the sample (Figure 1a). The TEM image shows the as-grown film, which consists of multiple BNNS clusters that overlap each other. Each BNNS cluster is highly transparent. A partial wrinkle in the nanosheets is also observed, which is possibly caused by perturbations induced by the scratching of the BNNSs from the sample to a template for TEM measurements.

Figure 1c shows the SEM of a thick and opaque BN layer/sheet prepared with a much longer deposition time. Detailed discussion on the difference between thick and thin BNNSs can be found in our previous paper [39]. Within the first 5 min of deposition, the obtained super-thin BNNSs appear to be highly transparent. Each BNNS with a precise shape is clearly visible, indicating a good crystalline structure. The long duration of synthesis yields thick BNNSs, but once beyond a critical layer thickness, which depends on stress/strain and the chemical potential of the deposited film, growth continues through the nucleation and coalescence of adsorbate ‘islands’, causing a more delicate influence on a structural transformation, and finally forming an opaque BN layer/sheet with random edge structures at the top surface of the sample. In most cases, such an opaque BNNS has poor crystalline structures. Clearly, the thickness of the obtained BNNSs prepared for 50 min exceeds the critical dimension that changes the growth mechanism, and forms a poor crystalline structure of the BNNSs. It should be mentioned that no obvious changes in the morphologies of the BNNS sample before and after plasma treatment are found from their SEM and TEM images. In the present cases, all of the plasma treatments are based on the low-temperature, low-power, radio-frequency (RF) plasma source. The detailed discussions of the plasma source and plasma effect are presented in the section regarding the plasma effect on the response of the photodetector.

### 2.3. Raman Analyses

**Figure 2:**
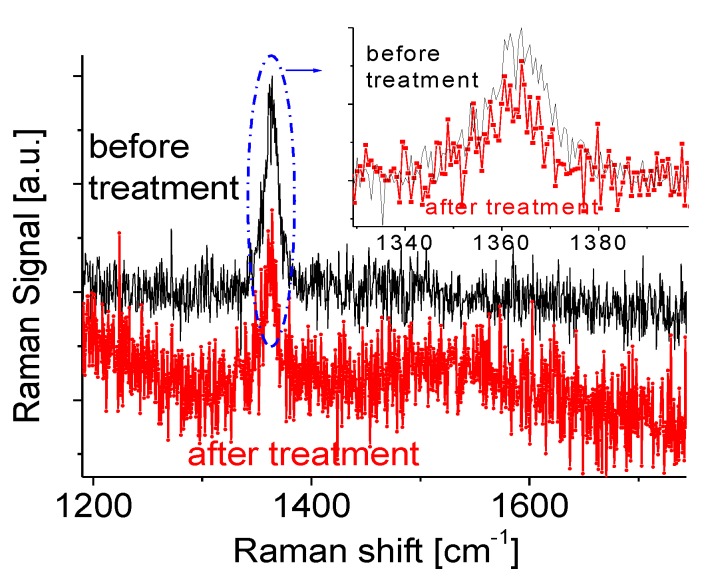
Raman scattering spectra of the BNNS sample (Figure 1a) before and after plasma treatments.

A Raman system (JEOL Ltd. JSM-6340F, Tokyo, Japan) with triple monochromators and an Ar^+^ ion laser (~2 mW with an excitation light of 514 nm) was used to characterize the Raman scattering spectra of the BNNSs. An Olympus microscope with an 80× microscope objective focuses the laser beam giving a spot diameter of 2–3 µm on the BNNS sample surface. The accumulation time is 30 s for the measurement. The sample surface morphology remains nearly unchanged before and after Raman spectral measurements, indicating there is no annealing effect with an accumulation time of 30 s. A detailed description of our Raman system was presented in our previous paper [17]. The Raman scattering data of the BNNSs are depicted in Figure 2.

A sharp Raman at 1366 cm^−1^ directly corresponds to the active E_2g_ mode of h-BN [40]. This narrow Raman spectral profile suggests that the pulse laser-produced plasma deposition technique yields high-quality BNNSs. High background noise is probably due to a short accumulation time in Raman measurement and super thin BNNSs. A good method for studies of crystalline structures of very thin samples is low-energy electron diffraction (LEED) spectroscopy. Detailed characterizations of highly-transparent BNNS and opaque BNNS samples can be found from our previous paper [41]. After plasma treatment, the Raman spectral line of BNNSs still appear to have a narrow peak. Two very small changes have been observed from the magnified spectral lines, as shown in the insert in Figure 2 after comparing two lines before and after the treatment. The first is the Raman active E_2g_ mode of the sample with the hexagonal phase shifts from 1365 cm^−1^ to 1362 cm^−1^. The second is that the Raman peak after the treatment appears to have a better symmetric profile, suggesting less contamination concentration or contamination effect on the treated BNNS sample. Slight asymmetry of the spectral profile before the treatment usually indicates the existence of small defect concentrations or contaminations in BNNSs. This has been confirmed with the experimental data obtained from XRD measurements. 

**Figure 3:**
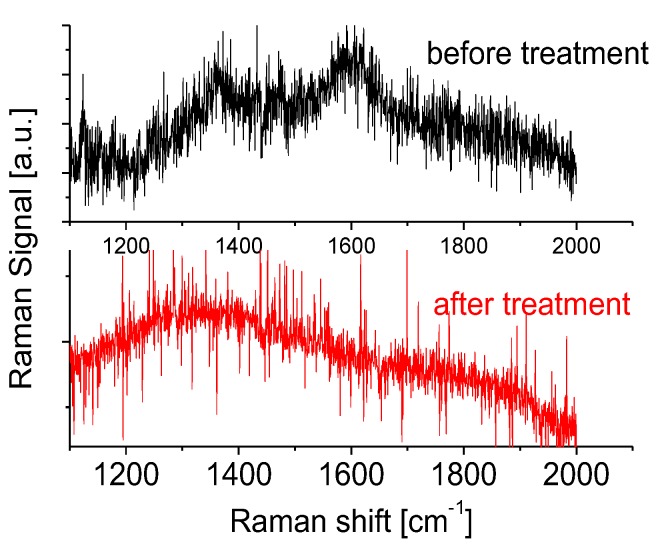
Raman scattering spectra of the BNNS sample (Figure 1c) before and after plasma treatment.

As a comparison, Raman measurements of a thick, opaque BNNS sample are also carried out, and the data are presented in Figure 3. A weak and broadened peak at 1361 cm^−1^ related to hBN is observed that provides direct evidence that these opaque BNNSs have a poor quality in their crystalline structures. Furthermore, a peak located at 1593 cm^−1^ related to the contribution from carbon is also observed. After plasma treatment, the carbon component on the surface of the sample has been removed or cleaned up, largely, whereas there is no obvious improvement of the Raman spectral profile related to the quality of the BNNS sample. Therefore, in the following sections, the main work on the development of DUV photodetectors is based on high-quality BNNSs samples (Figure 1a).

### 2.4. XRD Analyses

**Figure 4:**
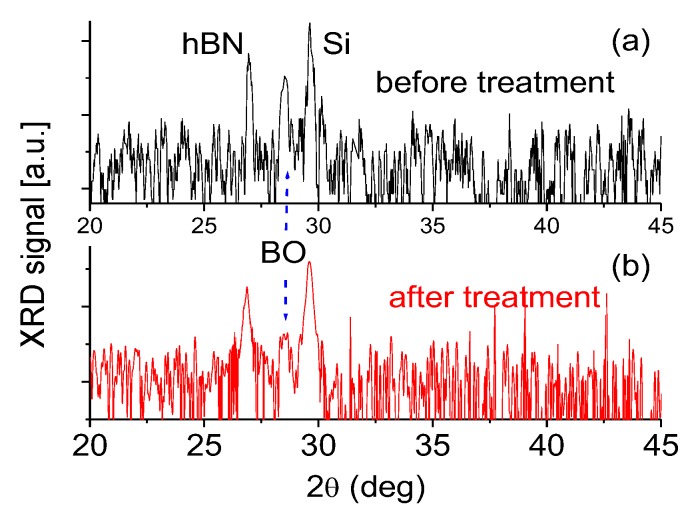
XRD spectra of the BNNS sample (Figure 1a) before and after plasma treatment.

Figure 4 shows a typical XRD of the first BNNS sample (Figure 1a) before and after plasma treatment. A strong XRD peak at 29.6° is related to the contribution of Si substrate. The peak at 2θ = 26.9° is assigned to hBN. A very small peak at 2θ = 28.6° is associated with a boron oxide (B_2_O_3_) component presented in the BNNSs. A small amount of oxygen is probably from the residual gas in the deposition chamber during synthesis or from the air during the transportation to characterization chambers. Plasma treatment can effectively remove most of the oxygen component from the sample. As a result, the XRD signal of the B_2_O_3_ component decreases significantly in its spectral intensity, as shown in Figure 4. This is in good agreement with the experimental data obtained from the Raman measurements. 

## 3. Results and Discussions

### 3.1. Fabrication of the Prototype and Characterization of Its Electrical Properties

To the best of our knowledge, almost all reported self-powered DUV photodetectors were based on the ZnO, TiO, or GaN materials [42,43,44,45,46,47], and little success has been made for the operation of BN-based self-powered DUV photodetectors. 

In order to develop high-performance, self-powered BNNS-based DUV photodetectors, a Schottky barrier is fabricated on high-quality crystalline BN nanosheets at first. The alignment of the Fermi level of the metal and the semiconductor gives a barrier for the electrons [48,49]. In the present case, the Schottky barrier is formed at the interface between the Au and the BNNS. Since active layer is extremely thin, it is expected that the entire region of the BNNS would be almost depleted. 

**Figure 5:**
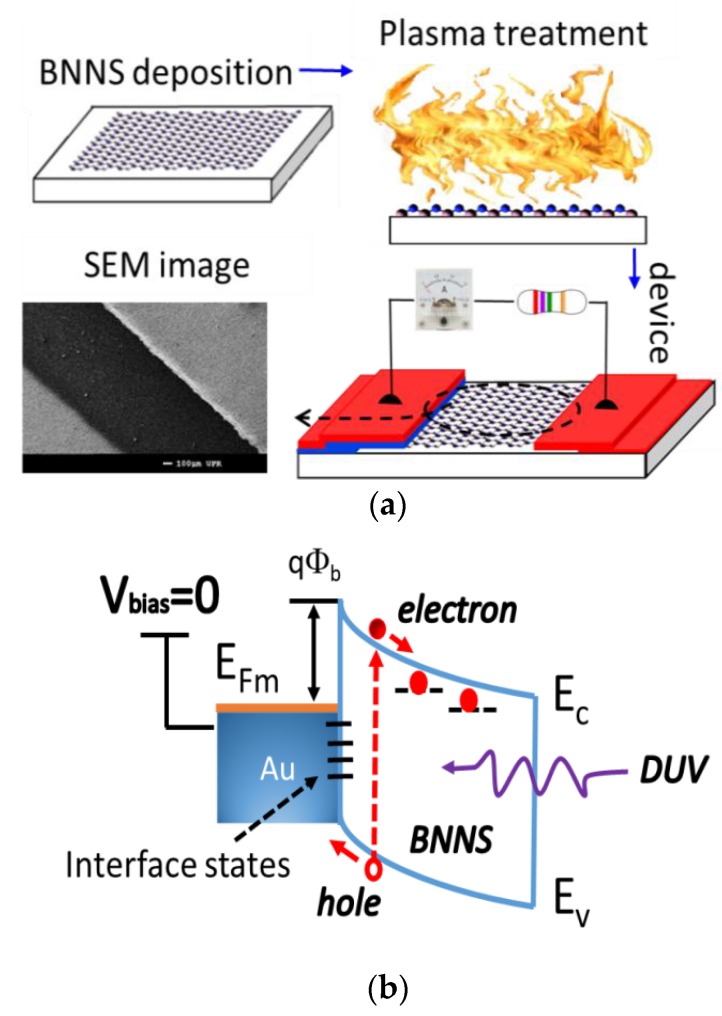
(**a**) Process flow for fabrication of self-powered BNNS-based DUV photodetectors; and (**b**) schematic energy band diagram of the Au–BNNS Schottky contact under UV light illumination.

The process flow for fabrication of self-powered BNNS-based DUV photodetectors is presented in Figure 5a. The BNNS-based membrane with a thickness around 0.3–0.4 µm is used as an active layer for the prototype. Au and Al electrodes 70–80 nm thick are deposited, respectively, onto two sides of the surface of the as-grown BNNS active layer using the plasma sputtering deposition technique. Then, another Au protective coating is deposited onto the Al electrode in order to avoid corrosion. The total exposure area of the active layer is 1.0 × 5 mm. The structure of the prototype is simple, and the fabrication is fast. After annealing at 200 °C for 30 min, the prototype is electrically characterized first, and followed by measurements of responsivity to DUV radiation.

Figure 5b shows a schematic band diagram of the Au–BNNS Schottky contact under UV light illumination. According to the Schottky-Mott theory, the Schottky barrier qΦ_b_ can be predicted from the difference between the work function of the metal and the affinity of the semiconductor. This barrier controls the current flow, but it can be modulated with an external voltage. Furthermore, the Schottky barrier is also likely to be a function of the interface atomic structure, and atomic heterogeneities at the metal-semiconductor interface, as well as the operating temperature [50,51]. For example, dark currents increase at higher measurement temperatures. Generally, drift current is the dominating current (dark current) in photoconductive (PC) mode and varies directly with the temperature. In photovoltaic (PV) mode, the diffusion current is the dominating current, which determines the shunt resistance. It varies with the square of the temperature. Thus, the change in temperature affects the photodetector more in photovoltaic mode than in the photoconductive mode of operation. The exact change relies on additional parameters, such as the applied reverse voltage, the bulk resistivity, dopant, concentration, and the thickness of the bulk substrate. Since the noise currents are generated as a result of dark current (shunt resistance), the higher the temperature, the higher the noise will be in the photodetector. The variation of the electrical properties of the Schottky barrier can be attributed to combined effects. 

**Figure 6:**
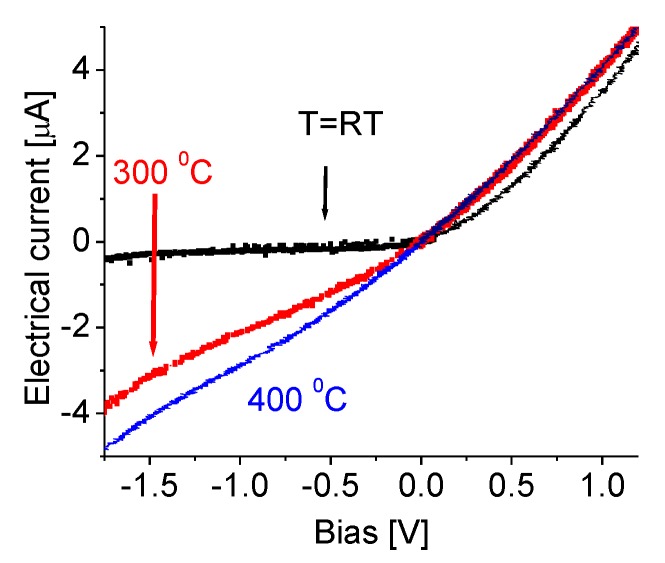
Current-voltage characteristics of the prototype at different temperatures.

The current-voltage (I–V) properties at different temperatures are characterized using an HP34401 (Keysight Technologies, Santa Rosa, CA, USA) and a Keithley 6517 A multimeter controlled (Keithley, Cleveland, OH, USA) via LabVIEW software (Keysight Technologies, Santa Rosa, CA, USA). The measurements are conducted in standard ambient conditions. Figure 6 presents the typical I−V characteristics of the prototype operating at 25, 300, and 400 °C, respectively. Nonlinear current-voltage curves are clearly visible. In the reverse bias region, there is only a very low reverse saturation current through the device at room temperature, indicating the backward current is largely blocked. In contrast, the forward current increases slowly, and then quickly, with an increase of the forward voltage. The complete I–V curve appears to have a typical behavior of a simple Schottky diode. Its tendency to conduct electric current is only in one direction. A variation of temperature significantly affects the backward current as seen in Figure 6, but forward current-voltage curves remain almost unchanged. This might suggest that high temperature would cause a decrease in the Schottky barrier, resulting in the increase of reverse electric current. A detailed discussion of doping, electron mobility, and concentration related charge carrier transport properties in layer structured hexagonal boron nitride can be found in Jiang’s papers [52,53]. In contrast to the electrical transport properties of traditional III-nitride wide band gap semiconductors, the temperature dependence of the hole mobility in hBN can be described by the form of μ ∞ (T/T_0_)^−α^ with α = 3.02, satisfying the two-dimensional (2D) carrier transport limit dominated by the polar optical phonon scattering. This behavior is a direct consequence of the fact that hBN is a layer-structured material. It should be mentioned here that the present work focuses on the high-temperature performance of self-powered UV photodetectors, which do not need a power supply or a bias voltage. Therefore, no characterizations of the I–V properties under different UV illuminations were conducted.

### 3.2. Response at Zero Bias to Various UV Wavelengths at Different Intensities

After fabrication, characterizations of the responses of the prototype to 250 nm UV light with different light intensities are carried out. The fabricated prototype operates in photovoltaic mode [8]. Therefore, the yielded dark current is weak, and the obtained signal-to-noise ratio is high.

**Figure 7:**
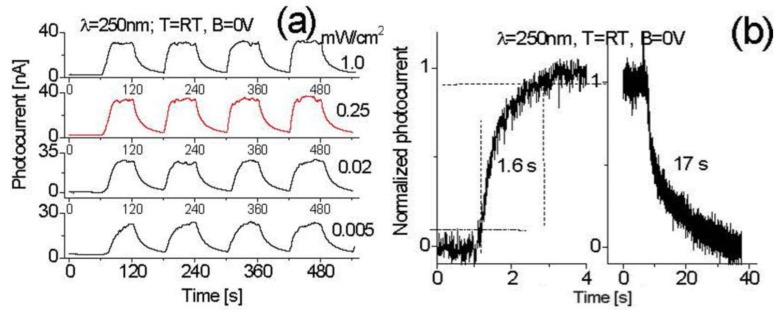
(**a**) Induced photocurrent responses at room temperature when the device is cycled with a period of 2 min between the “switch-on” and “switch-off” of 250 nm UV light at different intensities; and (**b**) the time response of the device to 250 nm radiation at 1 mW/cm^2^. RT: room temperature; λ: wavelength; B: bias.

Figure 7a shows the induced photocurrent responses at room temperature when the device is cycled with a period of 2 min between the “switch-on” and “switch-off” of 250 nm UV radiation at different intensities. Figure 7b shows time responses of the device to 250 nm exposure at an intensity of 1 mW/cm^2^. When the prototype is exposed to 250 nm radiation, the induced photocurrent quickly rises at first, and then reaches a stable value. When the UV light is turned off, the induced photocurrent quickly decreases, and then gradually decays to zero, as shown in Figure 7a. The induced photocurrent could be attributed to DUV photons absorption. The prototype has very good repeatability and stability features. The obtained maximum photocurrent is 34 nA and dark current is 2 nA, yielding a signal-to-noise ratio up to 17. Since the 250 nm light power on the active layer (exposed area: ~5 mm^2^) is 50 µW, the estimated sensitivity (total yielded photocurrent/total light power on the active layer) of the prototype is around 0.7 mA/W. This value is almost 78 times larger than the previously-reported results obtained either from BNNS-based or oxide semiconductor-based DUV photodetectors [23,47].

Following the decrease of 250 nm light intensity on the BNNS active layer of the prototype from 1.0 mW/cm^2^ to 0.25 mW/cm^2^, there is no obvious photocurrent decrease, but once the DUV light intensity drops to 0.02 and 0.005 mW/cm^2^, the generated photocurrent drops to 29 and to 23 nA, respectively. Correspondingly, a larger value of sensitivity (induced photocurrent/total incident light power) up to 80 mA/W is obtained. The phenomenon related to a small value of the sensitivity at intense light illumination is probably due to the saturation of light absorption.

It is found from Figure 7a that the response time of each cycle varies. The response times at the first few cycles are long, and then become short after the first several cycles are completed. This variation in time is dependent on many factors such as the DUV wavelengths, Schottky barriers, operating temperature, and environment humidity content, among others. This was discussed before in the case of SiC-based DUV photodetectors [17].

In order to analyze the change in response time, a high-resolution Cobox interface is used to re-measure the response time of the device. Typical results are presented in Figure 7b, from which the response time of the device can be estimated around 1.6 s and the recovery time around 17 s. The actual response time and recovery time might be shorter, because of the delay in reaching the full intensity after turning on the lamp, and the fluorescence after turning off the lamp. 

**Figure 8:**
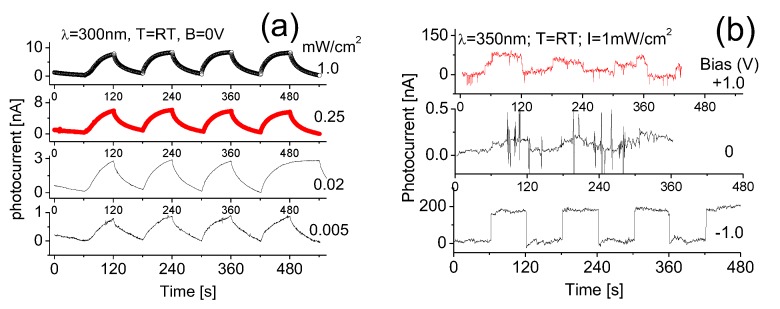
Induced photocurrent responses at room temperature, when the device is cycled with a period of 2 min between the “switch-on” and “switch-off” of (**a**) 300 nm, and (**b**) 350 nm UV light at different intensities. RT: room temperature; λ: wavelength; B: bias.

As a comparison, measurements of the induced photocurrent responses to 300 nm and 350 nm light are also performed and the results are shown in Figure 8a,b, respectively. Clearly, the response strength or yielded photocurrent from the device exposed to 300 nm radiation was weak, around foiur times less than that of 250 nm light at the same intensity, indicating the fabricated BNNS-based detector is more sensitive to a shorter UV wavelengths. The obtained response time is also slightly longer. These features could be directly attributed to BNNS material electronic properties such as large band gap width and band structures. No obvious cut off response is observed, although the response strength to 300 nm light is weak and its response time is long. 

Here it needs to be pointed out that in the last cycle the time duration for switch-on of the 300 nm UV radiation with the light intensity of 0.02 mW/cm^2^ is 2 min (120 s), in order to investigate the truly stable state of response. Clearly, after the rise time, the response strength at zero bias appears very stable, as shown in Figure 8a. However, the obtained photocurrent from the device to 350 nm UV light at 1 mW/cm^2^ irradiance is extremely weak, less than 0.5 nA, and the signal-to-noise ratio is poor. The weak light-induced photocurrent is largely merged with relatively strong background noise, but the light-induced photocurrent is still detectable, as shown in Figure 8b. 

Interestingly, a slight increase of the bias from zero to one voltage would effectively enhance the yield of photocurrent from the prototype. For example, the applied backward bias of 1 V would result in an induced photocurrent up to 180 nA. In contrast, the forward bias of 1 V generated an 80 nA photocurrent from the device exposed to 350 nm UV radiation at 1 mW/cm^2^. The forward bias normally causes strong dark current and noise. This is in good agreement with the results obtained from the I–V characterization above. 

### 3.3. Temperature Effect

**Figure 9:**
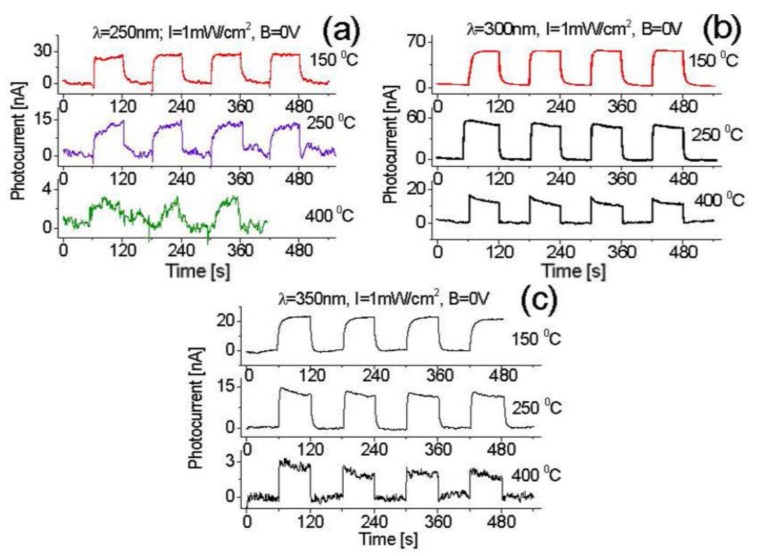
Thermal effect on the photocurrent response of the zero-biased device exposed to (**a**) 250; (**b**) 300; and (**c**) 350 nm UV radiation at a light intensity of 1 mW/cm^2^.

In addition to the bias effect, experiments are also carried out to investigate the temperature effect on the properties of BNNS-based DUV photodetectors. Several groups previously reported their achievements to have high-temperature DUV photodetectors based either on multi-layered oxide semiconductors [27] or on SiC material [17,28], but none of these reported detectors could operate well at a temperature above 200 °C. Therefore, it is necessary to study the high-temperature effect on new BNNS-based DUV photodetectors. Figure 9 shows the thermal effect on the photocurrent response of the device to (a) 250, (b) 300, and (c) 350 nm light at 1 mW/cm^2^ irradiance, respectively, in a photovoltaic mode. 

Compared with the responses at room temperature shown in Figure 7a, it is found that the obtained light-induced photocurrents at a large temperature range (25–150 °C) remain nearly unchanged, which indicates the performance of the newly-fabricated BNNS-based DUV photodetector is unperturbed by temperature in this range. This feature is due to the high-stability properties of boron nitride materials. 

A further increase of operating temperature to 250 °C, the thermal noise becomes visible, and the induced photocurrent drops to 15 nA. However, the prototype still runs well with excellent stability and repeatability features, as shown in Figure 9a. Those characteristics are obviously better than that of either oxide semiconductor-based or SiC-based photodetectors. However, once the operating temperature increases up to 400 °C, the thermal noise strength further increases, and the induced photocurrent decreases to 2.7–3.1 nA. When tested under 300 nm radiation, a much better response is observed that the induced photocurrent at the zero bias increases to 56.8 nA with an increase of the operating temperature to 150 °C, whereas the generated thermal noise is still extremely weak. These data suggest that an increase of the operating temperature would improve both device response and output signal-to-noise ratio at 300 nm wavelength, as shown in Figure 9b. Following the increase of temperature to 400 °C, the induced photocurrent reduces to 14.2 nA, but no obvious thermal noise is observed. It is found that a high operating temperature would also obviously improve the response time/rise time and recovery time. For example, at room temperature, the obtained rise time is around a few seconds (Figure 8b), but once the temperature is up to 400 °C, the rise time becomes much shorter, less than 300 ms (Figure 9b). The obtained experimental data clearly demonstrate that the BNNS-based DUV detector has excellent performance, including short response time, high induced photocurrent, high baseline stability, and good repeatability, in a wide temperature range. 

A similar phenomenon is also observed from the device exposed to 350 nm UV radiation. As shown in Figure 9c the response strength/induced photocurrent is up to 22.4 nA with an increase of temperature from 25 °C to 150 °C, and then decreases from 22.4 nA to 12.2 nA, and to 2.2 nA with a continuous increase of temperature from 150 °C to 400 °C. All parameters have been listed in Table 1.

It is also observed that an increase of the operating temperature would result in shortening the response time/rise time and recovery time. From the obtained experimental data above, we can conclude that the temperature effect is more obvious on the photocurrent induced at longer DUV wavelengths than that at shorter DUV wavelengths. For example, with a temperature change from 20 °C to 150 °C, the 250 nm light-induced photocurrent remains nearly unchanged around 34 nA. In contrast, 300 nm light-induced photocurrent increases almost 6–7 times from 9 nA to 56.8 nA, while 350 nm light-induced photocurrent increases almost 100 times from 0.14 nA to 22.4 nA. These changes are expected to be related to the band gap structures. 

### 3.4. Plasma Treatment

**Figure 10:**
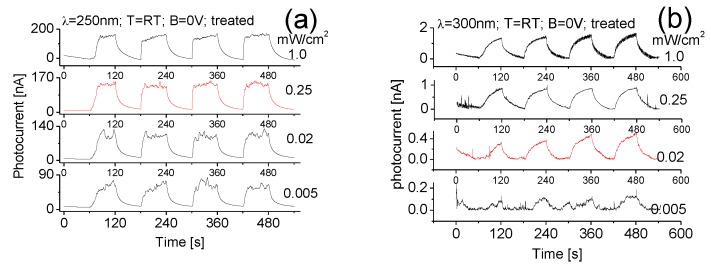

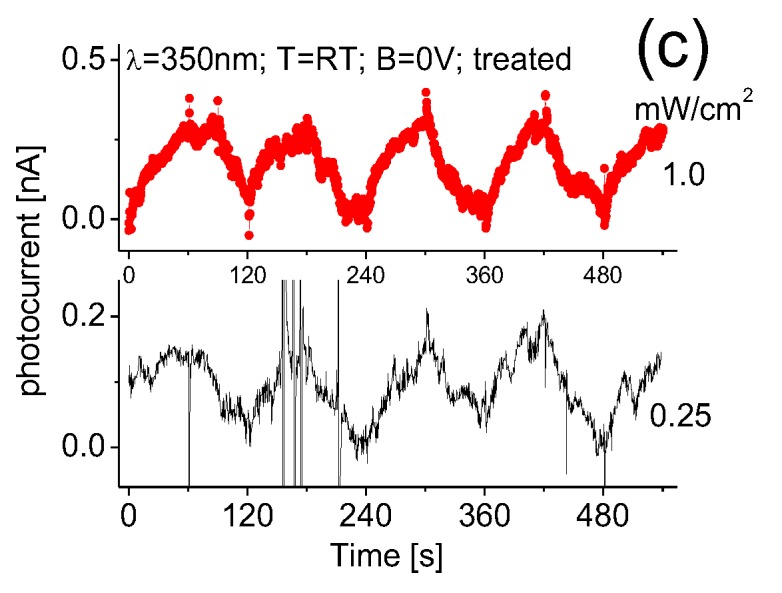
Responses of the prototype with plasma treated active layer to different intensities of (**a**) 250; (**b**) 300; and (**c**) 350 nm UV light. RT: room temperature; λ: wavelength. B: bias.

It has been found in the recent experiment that the plasma treatment would largely enhance the induced photocurrent of BNNS-based DUV photodetectors. An argon plasma source created at 100 mTorr pressure by a Harrick Plasma device is used to treat each BNNS sample for 40 min. In order to minimize possible damage by energetic ions, a low RF power (10–15 W) is applied to the coil to create a low-temperature plasma source. After the treatment, all measurements have been re-conducted. Figure 10 shows the typical induced photocurrent responses at room temperature when the device is cycled with a period of 2 min between the “switch-on” and “switch-off” light of (a) 250, (b) 300, and (c) 350 nm, at different intensities. 

Three differences in the responses or sensitivities of the device before and after plasma treatment can be easily distinguished. First, the induced photocurrent of the device exposed to 250 nm light at 1 mW/cm^2^ intensity at room temperature enhances from 34 nA to 162.7 nA, whereas its dark current falls to 0.4 nA, giving rise to a signal-to-noise ratio up to 350. This value is almost 20 times larger than that without the plasma treatment. Secondly, the estimated sensitivity of the prototype after plasma treatment up to 3.5 mA/W is obtained at saturation conditions (with 250 nm light at 1 mW/cm^2^), whereas the obtained maximal sensitivity at the condition without saturation can reach up to 296 mA/W. Finally, the prototype exposed to either 300 nm or 350 nm light appears to have a poor response strength and poor response time after the plasma treatment. As seen in Figure 10 the photocurrent generated by 300 nm light exposure at the same intensity declines from 9 nA before to 1.5 nA after the treatment, and the photocurrent created by 350 nm light is only 0.33 nA. Following the decrease of light intensities from 1.0 mW/cm^2^ to 0.25 mW/cm^2^, 0.03 mW/cm^2^, and then to 0.005 mW/cm^2^, the photocurrents corresponding to 250 nm illumination slightly decreases from 162 nA to 139 nA, 105 nA and then 67 nA. In contrast, the induced photocurrent by 300 nm light drops from 1.5 nA to 0.85 nA, 0.53 nA, and then 0.11 nA, as shown in Table 2. 

**Figure 11:**
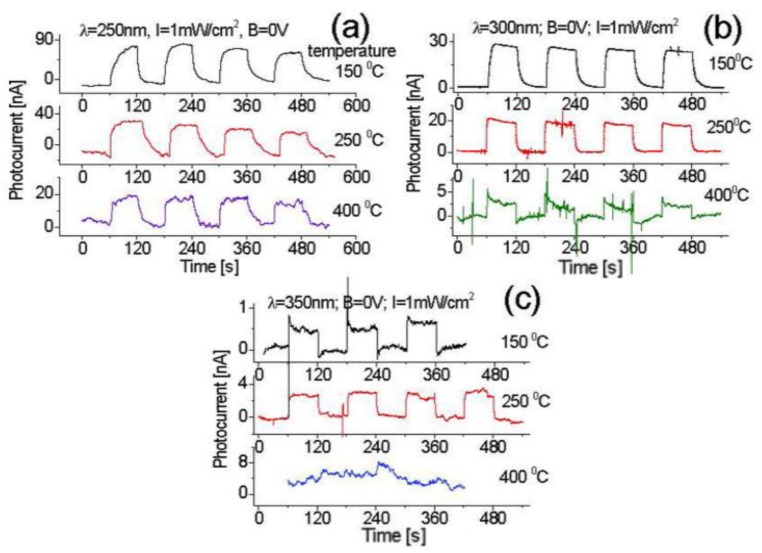
Temperature effect on the response of the zero-biased prototype exposed to (**a**) 250; (**b**) 300; and (**c**) 350 nm light at 1 mW/cm^2^ intensity after plasma treatment.

Different thermal effects on the induced photocurrent response of the prototype to 350 nm UV light after the plasma treatment are also characterized, and the results are shown in Figure 11. Experiments indicate the light-induced photocurrents are 81 nA for 250 nm light, 26 nA for 300 nm, and 0.64 nA for 350 nm at the operating temperature of 150 °C. Compared with the temperature effects on the response before the plasma treatment as shown in Figure 9 and Figure 11, 250 nm light-induced photocurrent increases around 3–4 times after plasma treatment at the same light intensity, but decreases down to 2.5 times for 300 nm light and 35 times for 350 nm. This is in good agreement with the result obtained from the comparison of data from Figure 7a, Figure 8 and Figure 10 at room temperature.

Following an increase of operating temperature from 150 °C to 400 °C, the thermal noise became slightly stronger, as seen in Figure 11a, while the induced photocurrent quasi-lineally drops from 162 nA at room temperature to 18 nA at 400 °C when exposed to 250 nm light. 

In contrast to the case with 300 nm light exposure, the induced photocurrent increases up to 26 nA at 150 °C at first, and then decreases down to 2.4 nA at 400 °C. Relatively, 350 nm light-induced photocurrent is weak. Following an increase of temperature from room temperature to 150 °C and then to 250 °C, 350 nm light-induced photocurrent increases from 0.3 nA to 0.6 nA, and then to 2.9 nA, but at the operating temperature up to 400 °C, no more signal can be detected as shown in Figure 11c. 

Summarizing the experimental data mentioned above, we may conclude that there is a shift in the cutoff wavelength in BNNSs synthesized using the pulse laser plasma deposition method. Many efforts have been conducted towards developing BN nanomaterial energy band gap width modulation techniques. Different orientations and edge structures of 2D BNNSs could result in different energy band gap widths from 5.5 eV down to 3.3 eV that have been recently experimentally proved by using cathode luminescence (CL) spectra [26].

Recent theoretical calculations also predicted two-dimensional (2D) materials, including BNNSs, would have a narrowed band gap and improved conductivity tuned by an external electric field [54], edge structures [55], and thickness [56]. One of the expectations is recently proved by measuring the electric and electronic properties of boron nitride nanoribbons via boron nitride nanotube unwrapping through plasma etching [57]. The theoretical calculation gives a valuable experimental implication: intriguing electrical and electronic properties or band gap width of the BNNSs can be precisely and effectively engineered by control of edge structures and electric fields [54,58], or even by strain [59].

Chemical (doping or impurity) manipulation is another powerful method to achieve controllable band gap structure-related electronic properties of 2D sheet materials [60,61]. For example, hydrogen treatments can effectively manipulate the band gap width of the BNNSs. Obvious red shifts of Raman spectral lines, X-ray diffraction peaks, and Fourier Transform Infra-red (FTIR) transmittance spectra were observed, respectively [62].

Since the present active layer is composed of a large amount of BNNS clusters with random orientations and different edge structures, it is possible to modify the band gap structures. Furthermore, a low concentration of impurity existing in the active layer would play a similar role as weak doping in BNNSs, resulting in a slightly manipulated band gap structure that improves the 300 nm light-induced photocurrent as shown in Figure 8a. An increase of operating temperature would further enhance impurity activity. Correspondingly, it would generate a stronger photocurrent by longer wavelength DUV light, as shown in Figure 9. However, once plasma treatment is performed, the impurity on the surface of the BNNSs would be largely removed as shown in Figure 4 that weakens the doping effect. As a result, either 300 nm or 350 nm light-induced photocurrent from the prototype would significantly decrease, whereas the 250 nm light-induced photocurrent increases. Based on Mendoza’s model [63], we could conclude that the band gap width shift to 5 eV (related to 250 nm light) is due to the sheet orientation distribution and its shift to 4.13 eV (related to 300 nm light) is because of impurity contributions. In fact, the feature of the band gap shift can also be characterized using a Tauc plot from the absorbance spectrum. Unfortunately, the direct absorption spectral measurement setup is not available in our laboratories. Although the general understanding is that the response strength is associated with the band gap electronic properties, the exact relationships between the thickness, orientations, and edge structures of BNNS materials and the band gap structure or width related cutoff wavelength shift are still not very clear. All those questions need to be studied further.

## 4. Conclusions

The experiments demonstrated that the BNNS-based self-powered DUV photodetector has excellent performance, including quick response time, large photocurrent, high baseline stability, and good repeatability within a wide temperature range up to 400 °C. At room temperature, the obtained signal-to-noise ratio is up 17. The obtained response before plasma treatment is up to 0.7 mA/W which is almost 78 times larger than that previously reported from either BNNS-based or oxide semiconductor-based DUV photodetectors. After plasma treatment, the obtained maximal response is 296 mA/W. 

It is found that the response strength of the device exposed to 300 nm light is almost 4–5 times less than that to 250 nm light, whereas 350 nm light-induced photocurrent is ~40 times less than that of 300 nm light at the same radiation intensity. When the temperature increases to 150 °C, the obtained 250 nm light-induced photocurrent remains nearly the same, indicating that the performance of the fabricated BNNS-based DUV photodetector is unperturbed by temperature in a wide range from 20 °C to about 150 °C. In contrast, the 300 nm light-induced photocurrent would increase up to 60 nA, almost two times higher than that with 250 nm light radiation. Similarly, 350 nm light-induced photocurrent increases up to 20 nA. 

It can be concluded that an increase of the operating temperature would improve both the device’s response and output signal-to-noise ratio at longer DUV wavelengths. When the operating temperature is up to 400 °C, the fabricated device still performs well in response strength, stability, and repeatability. It is also found that high operating temperatures would notably improve the rise time and decay time. The rise time becomes much shorter, less than 300 ms. 

It can also be concluded that plasma treatment could significantly enhance the 250 nm light-induced photocurrent from 34 nA to 150 nA, whereas its dark current decreases down to 0.4 nm, giving rise to a signal-to-noise ratio up to 350. This value is almost 20 times higher than the device without the treatment.

## Figures and Tables

**Table 1:** The induced photocurrents at different light intensities and temperatures.

**T = RT, before plasma treatment**					
	I (mW/cm^2^)	1	0.25	0.02	0.005
Light (λ)					
250 nm		34 nA	33 nA	29 nA	23 nA
300 nm		9 nA	5.5 nA	2.7 nA	0.8 nA
350 nm		0.14 nA	-	-	-
**I = 1 mW/cm^2^, before plasma treatment**					
	T (°C)	RT	150	250	400
Light (λ)					
250 nm		34 nA	30 nA	14 nA	2.7 nA
300 nm		9 nA	56.8 nA	53 nA	14.2 nA
350 nm		0.14 nA	22.4 nA	12.2 nA	2.2 nA

**Table 2:** The induced photocurrents at different light intensities and temperatures.

**T = RT, after plasma treatment**					
	I (mW/cm^2^)	1	0.25	0.02	0.005
Light (λ)					
250 nm		162.7 nA	139 nA	105 nA	67 nA
300 nm		1.5 nA	0.85 nA	0.53 nA	0.11 nA
350 nm		0.33 nA	0.18 nA	-	-
**I = 1 mW/cm^2^, after plasma treatment**					
	T (°C)	RM	150	250	400
Light (λ)					
250 nm		162.7 nA	81 nA	29 nA	18 nA
300 nm		1.5 nA	26 nA	17.8 nA	2.4 nA
350 nm		0.33 nA	0.64 nA	2.9 nA	-

## References

[1] Jiang H.X., Lin J.Y. (2014). Hexagonal boron nitride for deep ultraviolet photonic devices. Semicond. Sci. Technol..

[2] Tan C.-K., Borovac D., Sun W., Tansu N. (2016). Dilute-As AlNAs Alloy for Deep-Ultraviolet Emitter. Sci. Rep..

[3] Verma J., Islam S.M., Protasenko V., Kandaswamy P.K., Xing H., Jena D. (2014). Tunnel-injection quantum dot deep-ultraviolet light-emitting diodes with polarization-induced doping in III-nitride heterostructures. Appl. Phys. Lett..

[4] Sajjad M., Jadwisienczak W.M., Feng P. (2014). Nanoscale structure study of boron nitride nanosheets and development of deep-UV photo-detector. ACS Nanoscale.

[5] Xu X., Xu C., Hu J. (2014). High-performance deep ultraviolet photodetectors based on ZnO quantum dot assemblies. J. Appl. Phys..

[6] Lien W.-C., Tsai D.-S., Chiu S.-H., Senesky D.G., Maboudian R., Pisano A.P., He H. (2011). Low-temperature, ion beam-assisted SiC thin films with antireflective ZnO nanorod arrays for high-temperature photodetection. IEEE Electron Device Lett..

[7] Chen H., Liu K., Hu L., Al-Ghamdi A.A., Fang X. (2015). New concept ultraviolet photodetectors. Mater. Today.

[8] Sang L., Liao M., Sumiya M. (2013). A comprehensive review of semiconductor ultraviolet photodetectors: From thin film to one-dimensional nanostructures. Sensors.

[9] BenMoussa A., Soltani A., Schühle U., Haenen K., Chong Y.M., Zhang W.J., Dahal R., Lin J.Y., Jiang H.X., Barkad H.A. (2009). Recent developments of wide-bandgap semiconductor based UV sensors. Diam. Relat. Mater..

[10] Goldsman N. Silicon Carbide Electronics: Deep Ultraviolet Detectors: Design, Modeling and Fabrication. Proceedings of the NASA Early Stage Technology Workshop: Astrophysics & Heliophysics.

[11] Omnès F., Monroy E., Muñozc E., Reverchond J.-L., Morkoc H., Litton C.W. (2007). Wide bandgap UV photodetectors: A short review of devices and applications. Gallium Nitride Materials and Devices II, Proceedings of the SPIE, San Jose, CA, USA, 22–25 January 2007.

[12] Alenezi M.R., Henley S.J., Silva S.R.P. (2015). On-chip fabrication of high performance nanostructured ZnO UV detectors. Sci. Rep..

[13] Liu Z., Li F., Li S., Hu C., Wang W., Wang F., Lin F., Wang H. (2015). Fabrication of UV photodetector on TiO_2_/diamond film. Sci. Rep..

[14] Velazquez R., Aldalbahi A., Rivera M., Feng P. (2016). Fabrications and application of single crystalline GaN for high-performance DUV photodetectors. AIP Adv..

[15] Laksana C., Chen M.R., Liang Y., Tzou A.J., Kao H.L., Jeng E., Chen J., Chen H.G., Jian S.R. (2011). Deep-UV sensors based on SAW oscillators using low-temperature-grown AlN films on sapphires. IEEE Trans. Ultrason. Ferroelectr. Freq. Control.

[16] Dahal R., Li J., Majety S., Pantha B.N., Cao X.K., Lin J.Y., Jiang H.X. (2011). Epitaxially grown semiconducting hexagonal boron nitride as a deep ultraviolet photonic material. Appl. Phys. Lett..

[17] Aldalbahi A., Li E., Rivera M., Velazquez R., Altalhi T., Peng X., Feng P. (2016). A new approach for fabrications of SiC based photodetectors. Sci. Rep..

[18] Lin C.R., Wei D.H., BenDao M.K., Chen W.E., Liu T.Y. (2014). Development of High-Performance UV Detector Using Nanocrystalline Diamond Thin Film. Int. J. Photoenergy.

[19] Pace E., De Sio A. (2010). Innovative diamond photo-detectors for UV astrophysics. Memorie della Società Astronomica Italiana Supplement.

[20] Li C., Bando Y., Liao M., Koide Y., Golberg D. (2010). Visible-blind deep-ultraviolet Schottky photodetector with a photocurrent gain based on individual Zn_2_GeO_4_Zn_2_GeO_4_ nanowire. Appl. Phys. Lett..

[21] Li L., Lee P.S., Yan C., Zhai T., Fang X., Liao M., Koide Y., Bando Y., Golberg D. (2010). Ultrahigh-Performance Solar-Blind Photodetectors Based on Individual Single-crystalline In_2_Ge_2_O_7_ Nanobelts. Adv. Mater..

[22] Jiang X.-F., Weng Q., Wang X.-B., Li X., Zhang J., Golberg D., Bando Y. (2015). Recent Progress on Fabrications and Applications of Boron Nitride Nanomaterials: A Review. J. Mater. Sci. Technol..

[23] Rivera M., Velázquez R., Aldalbahi A., Zhou A.F., Feng P. (2017). High Operating Temperature and Low Power Consumption Boron Nitride Nanosheets Based Broadband UV Photodetector. Sci. Rep..

[24] Aldalbahi A., Feng P. (2015). Development of 2D boron nitride nanosheets UV photoconductive detectors. IEEE Trans. Electron Devices.

[25] Li J., Majety S., Dahal R., Zhao W.P., Lin J.Y., Jiang H.X. (2012). Dielectric strength, optical absorption, and deep ultraviolet detectors of hexagonal boron nitride epilayers. Appl. Phys. Lett..

[26] Yu J., Qin L., Hao Y., Kuang S., Bai X., Chong Y.-M., Zhang W., Wang E. (2010). Vertically aligned boron nitride nanosheets: Chemical vapor synthesis, ultraviolet light emission, and super hydrophobicity. ACS Nano.

[27] Zou R., Zhang Z., Liu Q., Hu J., Sang L., Liao M., Zhang W. (2014). High Detectivity Solar-Blind High-Temperature Deep-UV Photodetector Based on Multi-Layered (l00) Facet-Oriented β-Ga_2_O_3_ Nanobelts. Small.

[28] Chang W.-R., Fang Y.-K., Ting S.-F., Tsair Y.-S., Chang C.-N., Lin C.-Y., Chen S.-F. (2003). The hetero-epitaxial SiCN/Si MSM photodetector for high-temperature deep-UV detecting applications. IEEE Electron Device Lett..

[29] Zhang S., Lian G., Si H., Wang J., Zhang X., Wang Q., Cui D. (2013). Ultrathin BN nanosheets with zigzag edge: One-step chemical synthesis, applications in wastewater treatment and preparation of highly thermal-conductive BN–polymer composites. J. Mater. Chem. A.

[30] Khan M.H., Huang Z., Xiao F., Casillas G., Chen Z., Molino P.J., Liu H.K. (2015). Synthesis of large and few atomic layers of hexagonal boron nitride on melted copper. Sci. Rep..

[31] Wang X., Zhi C., Li L., Zeng H., Li C., Mitome M., Golberg D., Bando Y. (2011). Chemical blowing of thin-walled bubbles: High-throughput fabrication of large-area, few-layered BN and Cx-BN Nanosheets. Adv. Mater..

[32] Du M., Wu Y., Hao X. (2013). A facile chemical exfoliation method to obtain large size boron nitride Nanosheets. CrystEngComm.

[33] Wang Y., Shi Z., Yin J. (2011). Boron nitride nanosheets: large-scale exfoliation in methanesulfonic acid and their composites with polybenzimidazole. J. Mater. Chem..

[34] Deepika, Li L.H., Glushenkov A.M., Hait S.K., Hodgson P., Chen Y. (2014). High-efficient production of boron nitride nanosheets via an optimized ball milling process for lubrication in oil. Sci. Rep..

[35] Lee C., Li Q., Kalb W., Liu X.-Z., Berger H., Carpick R.W., Hone J. (2010). Frictional characteristics of atomically thin sheets. Science.

[36] Zhi C., Bando Y., Tang C., Kuwahara H., Golberg D. (2009). Large-scale fabrication of boron nitride nanosheets and their utilization in polymeric composites with improved thermal and mechanical properties. Adv. Mater..

[37] Lei W., Potehauh D., Liu D., Qin S., Chen Y. (2013). Porous boron nitride nanosheets for effective water cleaning. Nat. Commun..

[38] Sajjad M., Peng X., Chu J., Zhang H., Feng P. (2013). Design and installation of a CO_2_-pulsed laser plasma deposition system for the growth of mass product nanostructures. J. Mater. Res..

[39] Feng P., Li E.Y., Sajjad M., Aldalbahi A., Chu J. (2015). Boron Nitride Nanosheets and Their Electrical Tunneling Effect. Sci. Adv. Mater..

[40] Sajjad M., Morell G., Feng P. (2013). Advance in Novel Boron Nitride Nanosheets to Nanoelectronic Device Applications. ACS Appl. Mater. Interfaces.

[41] Sajjad M., Ahmadi M., Guinel M.J.-F., Lin Y., Feng P. (2013). Large-scale synthesis of single-crystal and polycrystalline boron nitride nanosheets. J. Mater. Sci..

[42] Chen H., Yu P., Zhang Z., Teng F., Zheng L., Hu K., Fang X. (2016). Ultrasensitive Self-Powered Solar-Blind Deep-Ultraviolet Photodetector Based on All-Solid-State Polyaniline/MgZnO Bilayer. Small.

[43] Lin P., Yan X., Zhang Z., Shen Y., Zhao Y., Bai Z., Zhang Y. (2013). Self-Powered UV Photosensor Based on PEDOT:PSS/ZnO Micro/Nanowire with Strain-Modulated Photoresponse. ACS Appl. Mater. Interfaces.

[44] Zu X., Wang H., Yi G., Zhang Z., Jiang X., Gong J., Luo H. (2015). Self-powered UV photodetector based on heterostructured TiO_2_ nanowire arrays and polyaniline nanoflower arrays. Synth. Met..

[45] Xie Y., Wei L., Li Q., Chen Y., Yan S., Jiao J., Liu G., Mei L. (2014). High-performance self-powered UV photodetectors based on TiO_2_ nano-branched arrays. Nanotechnology.

[46] Prakash N., Singh M., Kumar G., Barvat A., Anand K., Pal P., Singh S.P., Khanna S.P. (2016). Ultrasensitive Self-powered large area planar GaN UV-photodetector using reduced graphene oxide electrodes. Appl. Phys. Lett..

[47] Chen X., Liu K., Zhang Z., Wang C., Li B., Zhao H., Zhao D., Shen D. (2016). Self-Powered Solar-Blind Photodetector with Fast Response Based on Au/β-Ga_2_O_3_ Nanowires Array Film Schottky Junction. ACS Appl. Mater. Interfaces.

[48] Yang Y., Guo W., Qi J., Zhao J., Zhang Y. (2010). Self-powered ultraviolet photodetector based on a single Sb-doped ZnO nanobelt. Appl. Phys. Lett..

[49] Lee B., Kim C., Lee Y., Lee S., Kim D.Y. (2015). Dependence of photocurrent on UV wavelength in ZnO/Pt bottom-contact Schottky diode. Curr. Appl. Phys..

[50] Kumar A., Latzel M., Christiansen S., Kumar V., Singh R. (2015). Effect of rapid thermal annealing on barrier height and 1/f noise of Ni/GaN Schottky barrier diodes. Appl. Phys. Lett..

[51] Chawanda A., Coelho S.M.M., Auret F.D., Mtangi W., Nyamhere C., Nel J.M., Diale M. (2012). Effect of thermal treatment on the characteristics of iridium Schottky barrier diodes on n-Ge (1 0 0). J. Alloys Compd..

[52] Doan T.C., Li J., Lin J.Y., Jiang H.X. (2014). Charge carrier transport properties in layer structured hexagonal boron nitride. AIP Adv..

[53] He B., Qiu M., Yuen M.F., Zhang W.J. (2014). Electrical properties and electronic structure of Si-implanted hexagonal boron nitride films. Appl. Phys. Lett..

[54] Zhang Z., Guo W., Yakobson B.I. (2013). Self-modulated band gap in boron nitride nanoribbons and hydrogenated sheets. Nanoscale.

[55] Park C.-H., Louie S.G. (2008). Energy gaps and stark effect in boron nitride nanoribbons. Nano Lett..

[56] Kang J., Zhang L., Wei S.H. (2016). A Unified Understanding of the Thickness-Dependent Bandgap Transition in Hexagonal Two-Dimensional Semiconductors. J. Phys. Chem. Lett..

[57] Zeng H., Zhi C., Zhang Z., Wei X., Wang X., Guo W., Bando Y., Golberg D. (2010). White graphenes: Boron nitride nanoribbons via boron nitride nanotube unwrapping. Nano Lett..

[58] Yamijala S.S.R.K.C., Pati S.K. (2014). Effects of edge passivations on the electronic and magnetic properties of zigzag boron-nitride nanoribbons with even and odd-line stone-wales (5–7 pair) defects. Indian J. Phys..

[59] Qi J., Qian X., Qi L., Feng J., Shi D., Ju L. (2012). Strain-engineering of band gaps in piezoelectric boron nitride nanoribbons. Nano Lett..

[60] Koswattage K.R., Shimoyama I., Baba Y., Sekiguchi T., Nakagawa K. (2011). Selective adsorption of atomic hydrogen on a h-BN thin film. J. Chem. Phys..

[61] Zhang H.X., Feng P.X. (2012). Controlling bandgap of rippled hexagonal boron nitride membranes via plasma treatment. ACS Appl. Mater. Interfaces.

[62] Feng P., Sajjad M., Li E.Y., Zhang H., Chu J., Aldalbahi A., Morell G. (2014). Fringe structures and tunable bandgap width of 2D boron nitride Nanosheets. Beilstein J. Nanotechnol..

[63] Mendoza F., Makarov V., Hildalgo A., Weiner B., Morell G. (2011). Solar-blind field-emission diamond ultraviolet detector. J. Appl. Phys..

